# Microalgal cell factories for bioenergy and climate action: a circular bioeconomy roadmap

**DOI:** 10.1186/s12934-026-03090-y

**Published:** 2026-09-29

**Authors:** Rehab H. Mahmoud, Hassan M. M. Mustafa, Ilunga Kamika, Fagr Kh. Abdel-Gawad, Ola M. Gomaa

**Affiliations:** 1 Water Pollution Research Department, National Research Centre, Dokki, Giza, 12622 Egypt; 2 Centre of Excellence for Research and Applied Studies On Climate Change and Sustainable Development (C3SD-NRC), National Research Centre, Dokki, Giza, 12622 Egypt; 3 Mechanical Engineering Department, Engineering and Renewable Energy Research Institute, National Research Centre, Giza, 12622 Egypt; 4 Institute for Nanotechnology and Water Sustainability (iNanoWS), School of Science, College of Science, Engineering and Technology, University of South Africa, Johannesburg, South Africa; 5 Radiation Microbiology Department, National Center for Radiation Research and Technology, Egyptian Atomic Energy Authority, Cairo, Egypt

**Keywords:** Microalgae biomass, Biofuels, Bioenergy, CO_2_ sequestration, Climate change, Wastewater treatment

## Abstract

Microalgae represent a crucial, multifunctional microbial cell factory platform for renewable energy and climate change mitigation. Their notably high lipid content (often exceeding 70% DCW) and rapid growth rates, which significantly surpass those of traditional terrestrial crops, making them an ideal feedstock for diverse bioenergy applications. This review critically evaluates advanced conversion pathways for biofuels such as biodiesel and bioethanol, as well as biohydrogen production and bioelectricity generation via microbial fuel cell technology. It further explores key technical paradigms, including photobioreactor and open-pond cultivation systems, extraction techniques, and the critical role of transesterification for biodiesel synthesis. Uniquely, microalgae synergistically sequester atmospheric carbon dioxide (CO_2_) during the photosynthetic process and remediate nutrient-rich wastewater, positioning them as an effective feedstock model within a circular bioeconomy framework. Finally, this work delineates persistent techno-economic hurdles, such as high operational costs and harvesting inefficiencies, while proposing strategic future research directions in genetic engineering and system integration necessary to achieve global commercial scalability.

**Graphical Abstract:**

**Figure:**
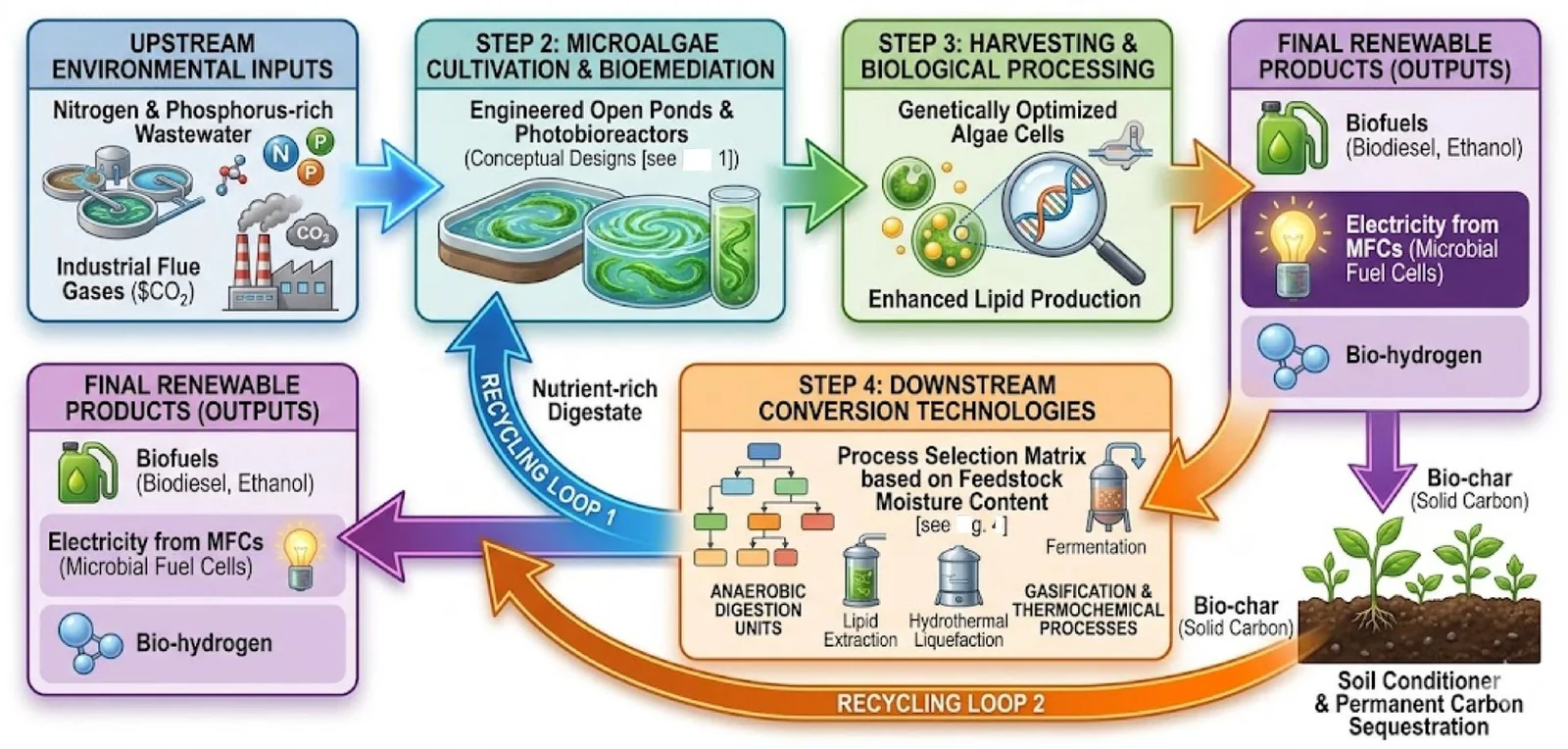

## Introduction

The escalating impacts of global climate change have rendered the transition from fossil fuels to sustainable energy sources an urgent imperative [1]. Fossil fuels combustion for power and heat generation accounts for over three-quarters of global greenhouse gas (GHG) emissions, with CO_2_ identified as the predominant contributor [2]. This has precipitated a critical need to decarbonize the global energy system, aligned with ambitious targets to halve emissions by 2030 and achieve net-zero by 2050 [3]. Among the promising renewable energy technologies, biomass-derived biofuels offer a viable pathway to displace carbon- intensive fuels [4, 5]. Microalgae, in particular, present a compelling alternative to first-generation biofuel feedstocks, which often compete with food production for arable land and freshwater [6]. These photoautotrophic microorganisms are distinguished by their rapid growth rates, and high biomass productivity, flourishing in diverse environments including non-arable and marginal waters [7].

The unique advantage of microalgae lies in their inherent efficiency at photosynthetic CO_2_ sequestration. This creates a dual benefit: providing a sustainable bioenergy feedstock while directly mitigating atmospheric GHG [8]. Certain species can accumulate lipid, which makes them prime candidates for biodiesel and biohydrogen production [9]. Furthermore, their cultivation can be integrated with industrial processes by utilizing flue gases for CO₂ and wastewater for nutrients, thereby advancing a circular bioeconomy model [10, 11]. Within this framework, we introduce a circular bioeconomy roadmap: an integrated, multi-stage system that closes material loops by utilizing waste streams, CO₂-laden flue gas, and nutrient-rich wastewater, as inputs for microalgae cultivation, and then sequentially converts all biomass fractions into bioenergy and co-products, generating minimal residual waste.

To achieve this, the proposed Circular Bioeconomy Roadmap transcends the ‘Process Lock-in’ challenge by advocating for Dynamic Biorefineries. Unlike traditional linear models, this roadmap integrates upstream engineering with downstream conversion to avoid the ‘Energy Trap’, the massive energy penalty of dewatering and drying. By shifting away from standalone systems and instead coupling large-scale cultivation with industrial infrastructures (e.g., utilizing municipal wastewater streams and industrial flue gas), the costs and resources needed for contamination management and mixing energy are offset by ‘feedstock credits,’ turning waste streams into economic drivers for scalable operations.

This review systematically examines the spectrum of microalgae-based bioenergy, providing an in-depth analysis of advanced conversion technologies. Recent breakthroughs in cultivation and process optimization are synthesized, alongside a critical evaluation of economic viability and life-cycle impacts. Finally, the current status and projected demand for algal bioenergy are highlighted, concluding with a forward-looking perspective on the key challenges and strategic research directions required for widespread commercial adoption.

Beyond reviewing these components individually, this review explicitly frames them as functionally interdependent stages of a single operating system. Industrial flue gas and nutrient-rich wastewater are introduced as nutrient inputs for the microalgae cultivation stage, mechanistically coupling the carbon-capture and wastewater-treatment functions of microalgae to biomass production rather than treating them as separate benefits. The harvested biomass is then not directed toward a single conversion route but processed through a cascade sequence: high-value proteins, vitamins and pigments are recovered first, lipid-rich fractions are directed to transesterification or hydrothermal liquefaction, and the remaining lipid-extracted and carbohydrate-rich residues are routed to anaerobic digestion or fermentation, with process water and nutrient-laden digestate recirculated back to cultivation. This sequential arrangement, cultivation, carbon capture and wastewater remediation, cascade biomass conversion, and co-product and nutrient recovery, constitutes the practical circular-bioeconomy system referred to throughout this review, and each of the following sections is organized to show how it contributes to this integrated system rather than as an isolated technology.

## Microalgae diversity and physiological potential

The immense phylogenetic and metabolic diversity of microalgae, spanning eukaryotic green algae, diatoms, eustigmatophytes, and prokaryotic cyanobacteria, offers a vast, largely untapped resource for bioenergy applications. Each taxonomic group possesses distinct physiological and biochemical traits, such as specialized lipid profiles, carbon storage mechanisms (notably starch and triacylglycerols), and varying environmental tolerances. These characteristics can be strategically harnessed for targeted biofuel production [12]. Furthermore, recent breakthroughs in genetic engineering and cultivation science are increasingly unlocking this metabolic potential, enabling the design of ‘designer strains’ optimized for high-yield, industrial-scale bioenergy systems.

### Clustered regularly interspaced short palindromic repeats (CRISPR)-based strain improvement in microalgae

To enhance microalgae productivity and broaden industrial applications, recent advances in genetic engineering have become increasingly important for microalgae as sustainable cell factories. CRISPR/Cas-based genome editing, particularly Cas9 and Cas12a, has enabled more precise manipulation of genes associated with lipid accumulation, carbon utilization, and stress tolerance, although species-specific delivery and low editing efficiency remain important challenges [13, 14]. In parallel, metabolic pathway engineering has been used to redirect carbon flux toward biofuel precursors and other high-value metabolites, improving both productivity and process flexibility [15]. Moreover, synthetic biology tools, including modular gene expression systems, promoter optimization, and chassis development, are facilitating the construction of designer strains with improved biomass productivity and industrial robustness. Together, these approaches provide a strong foundation for next-generation microalgae biorefineries. These engineering strategies complement advances in cultivation and downstream processing, strengthening the feasibility of next-generation microalgae biorefineries.

#### Key taxonomic groups and their strategic advantages

#### Chlorophyta (green algae)

Species such as *Chlorella vulgaris*, *C. sorokiniana*, and *Scenedesmus obliquus* are foundational to algal biofuel research due to their rapid growth rates and robust cultivation in both open ponds and photobioreactors [16]. Their primary advantage lies in their capacity to accumulate substantial triacylglycerides (TAGs) often exceeding 50% of dry cell weight under nutrient stress, making them ideal feedstocks for biodiesel production via transesterification.

##### Bacillariophyta (diatoms)

Diatoms, including *Phaeodactylum tricornutum* and *Thalassiosira pseudonana*, are notable for their ecological dominance and high natural productivity. They can store significant lipids and synthesize valuable co-products such as the omega-3 fatty acid eicosapentaenoic acid (EPA). Their silica-based cell walls (frustules) can facilitate harvesting, and many species are mixotrophic, capable of boosting biomass yields by assimilating organic carbon [17].

##### Cyanobacteria (blue-green algae)

Although typically low in lipid content, cyanobacteria like *Synechocystis* spp. and *Synechococcus* spp. offer a distinct advantage as platforms for synthetic biology. Their relatively simple prokaryotic genomes make them highly amenable to genetic modification for the direct photosynthetic production and secretion of hydrocarbons, alcohols, or free fatty acids, potentially bypassing energy-intensive biomass processing steps [18]. The filamentous cyanobacterium *Arthrospira platensis* (*Spirulina*) is also valued for its exceptionally high biomass productivity and ease of harvest.

##### D. Eustigmatophytes

*Nannochloropsis* spp., often classified separately from green algae, are considered leading industrial candidates. They exhibit a resilient physiology characterized by a robust cell wall that confers resistance to contamination and grazing in open systems. Crucially, they channel photosynthetic carbon preferentially into lipid rather than starch synthesis, enabling high oil content (frequently > 50% DCW) without severe growth trade-offs, a key attribute for economic viability [19].

#### Strain improvement: engineering the biological toolbox

Under optimal growth conditions, conventional microalgae typically accumulate lipids in the range of 15–30% of dry cell weight (DCW), a level often insufficient for commercial biofuel production. To bridge this gap, advanced biotechnological strategies including mutagenesis, adaptive laboratory evolution, and synthetic biology are being deployed to develop strains with lipid content exceeding 50–70% DCW (e.g., up to ~ 60% DCW in nitrogen-starved *Nannochloropsis* sp. flat-panel photobioreactor cultures, and reported values approaching 70% DCW in select laboratory-evolved oleaginous strains [20], alongside enhanced carbon fixation rates and substrate flexibility [21]. Beyond single-strain cultivation, the use of defined microalgae consortia is gaining traction. These multi-species communities drastically improve system stability and biological resilience to contamination through ecological niche partitioning and synergistic interactions, thereby offering a more robust, self-defending platform for large-scale cultivation [22]. The selection of a strain or consortium must be a holistic decision, balancing raw yield metrics (lipid/carbohydrate content) against cultivation robustness, harvestability, and co-product potential. This biological potential sets the stage for the engineering challenge: selecting and optimizing a cultivation system capable of translating these traits into sustainable, large-scale biomass production.

## Cultivation strategies and carbon sequestration: an engineering-environmental nexus

**Fig. 1:**
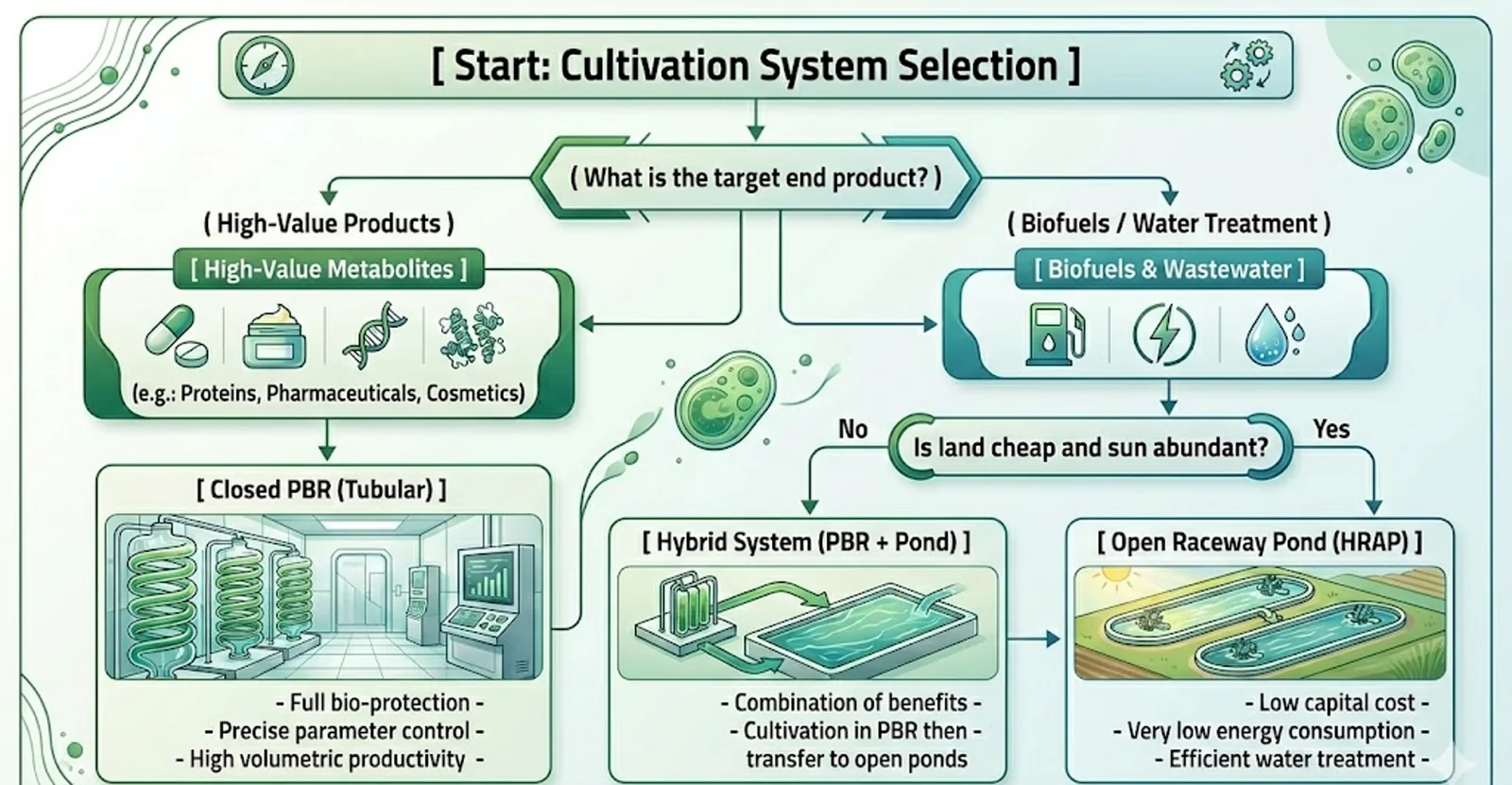
Engineering decision tree for microalgae upstream cultivation architecture based on geographic and economic trade-offs

The evolution of microalgae cultivation reflects a persistent search for equilibrium between scalability, cost-efficiency, and biological precision. Historically rooted in wild stock harvesting, modern systems now span a technological spectrum, from low-intensity open ponds to sophisticated, high-density photobioreactors (PBRs) [23]. Each configuration presents inherent trade-offs (Fig. 1; Table 1) that dictate its suitability for specific bioenergy pathways.

**Table 1:** Comparative Techno-economic and Performance Metrics of Open Raceway Ponds and Tubular Photobioreactors

Parameter	Open Pond (Raceway)	Photobioreactor (Tubular)	Ref.
Capital Cost (CAPEX)	$10 - $25 m^-2^	$100 - $250 m^-2^	[36]
Operating Cost (OPEX)	3.42–4.13 kg^-1^	3.54–5.78/kg	[37]
Cost per kg biomass	~ 5.00	~ 12.00	[38]
Vol. Productivity (g L⁻¹ d⁻¹)	0.1–0.5	1.0–2.5	[5]
Areal Productivity (g m⁻² d⁻¹)	15–25	30–50	[39]
Mixing & Operational Energy Input	0.1–2.1 W m⁻³	10–50 W m⁻³	[40]
Mass Transfer CO_2_ Coefficient (k_L_ a, h^-1^)	0.1–0.5 h⁻¹	1.8–3.9 h⁻¹	[41]
Utilization CO_2_ Efficiency (%)	20–50%	60–80%	[42]
Contamination Risk & Biosecurity	Extreme (Highly vulnerable to local ciliates, protozoa, and wild strains)	Minimal (Closed system providing strict biosecurity boundaries)	[43]
Process Control & Parametric Regulation	Non-automated; subject to ambient diurnal and seasonal variations	Highly automated; precise control over pH, temperature, and dissolved oxygen	[44]

*CAPEX = Capital Expenditure ($ m⁻²)

*OPEX = Operating Expenditure ($ kg⁻¹ biomass)

### Open and closed systems: the efficiency vs. cost paradigm

Open raceway ponds remain the primary vehicle for bulk biomass production due to their manageable capital expenditures (CAPEX) and operational simplicity. However, their economic appeal is often undermined by a “productivity ceiling” (10–25 g m⁻² d⁻¹), an areal range typically reported for species such as *Chlorella* and *Scenedesmus* in outdoor open raceway ponds under ambient irradiance and temperature, expressed as ash-free dry weight per unit pond area per day [24]. This limitation is intrinsically an engineering bottleneck driven by poor mixing dynamics, which induce the formation of stagnant dead zones within the pond architecture. Consequently, these mixing inefficiencies exacerbate mutual shading, forcing cells trapped in deeper layers to perish in prolonged darkness, while surface-layer cells are simultaneously subjected to severe photoinhibition. Furthermore, high evaporative losses and vulnerability to invasive “weed” species necessitate robust, high-alkalinity strains to maintain culture purity. To mitigate these biological contamination risks at scale, operational strategies must rely on strict chemical regimes. Selecting robust, high-alkalinity or high-salinity tolerant strains, such as *Chlorella*, *Scenedesmus*, or *Dunaliella*, allows the target culture to effectively outcompete invading contaminants and predatory organisms that cannot survive under such extreme environmental constraints. For example, techno-economic modeling of a 100-hectare open-pond facility in southern Spain projected a current biomass production cost of approximately €3.4 kg⁻¹ (≈ US$3.7 kg⁻¹), with process intensification expected to lower this toward €0.5 kg⁻¹ within a decade [25]. While optimized facilities can produce biomass at approximately 5.50–6.60 kg^-1^, this remains significantly above the threshold required for standalone biofuel competitiveness without the integration of high-value co-product valorization [28].

In contrast, closed PBRs are engineered to maximize photosynthetic efficiency by optimizing the surface-to-volume ratio. By enabling precise control over dissolved CO_2_ concentrations, pH, and thermal gradients, PBRs can achieve volumetric productivities of approximately 1–4 g m⁻² d⁻¹ (reaching up to ~ 4.2–4.3 g L⁻¹ d⁻¹ in optimized thin-layer cascade systems), several-fold higher than the equivalent volumetric output of open ponds, in tubular and flat-panel photobioreactors operating under controlled irradiance and temperature [29]. However, scaling up these systems introduces severe mass transfer limitations that trigger substantial economic and operational trade-offs. Achieving adequate CO_2_ mass transfer requires continuous micro-bubble sparging, which imposes an intense energetic penalty. Concurrently, large-scale geometries restrict effective gas degassing, leading to the toxic accumulation of photosynthetic oxygen; this accumulation induces severe oxidative stress within the culture and directly inhibits Rubisco activity, thereby bottlenecking overall photosynthetic efficiency. The sterile environment of PBRs is essential for cultivating sensitive “designer strains” engineered for specific lipid or carbohydrate profiles. Nevertheless, the high energy demand for cooling and the aforementioned accumulation of photosynthetic oxygen present significant operational hurdles that restrict their use to high-margin bioproducts [32].

#### Wastewater-based cultivation and nutrient recovery

As large-scale cultivation systems face persistent cost and contamination challenges, integrating wastewater as a nutrient source has emerged as a promising strategy to improve both economic and environmental performance. As large-scale cultivation systems face persistent cost and contamination challenges, integrating wastewater as a nutrient source has emerged as a promising strategy to improve both economic and environmental performance [33, 34]. Integrating wastewater into microalgae cultivation can substantially improve the economic viability of large-scale systems by supplying nutrients and reducing dependence on synthetic fertilizers [35, 36]. Reported optimized systems have achieved high nitrogen and phosphorus removal efficiencies, typically yielding total nitrogen (N) removal of 75%–92% and total phosphorus (P) removal of 80%–98% within a 5–7 day retention cycle (e.g., reducing total nitrogen from 45 mg/L to < 5 mg/L, and total phosphorus from 8 mg/L to < 0.5 mg/L), although performance varies with reactor configuration, hydraulic retention time, and wastewater characteristics [33]. However, wastewater-based cultivation introduces important trade-offs, including changes in biomass composition and increased biosecurity risks. Elevated ammonium availability can promote rapid growth and protein accumulation, but it may suppress lipid synthesis; therefore, a two-stage cultivation strategy is often recommended, where cells are first grown under nutrient-replete conditions and then exposed to nutrient stress to enhance lipid accumulation for biodiesel production. In addition, contamination by indigenous microorganisms and protozoa remains a major operational challenge, making non-sterile cultivation systems and stable microalgae-bacteria consortia more realistic than large-scale sterilization [37, 38]. Furthermore, co-accumulation of heavy metals may limit the application of the harvested biomass to thermal conversion pathways, such as hydrothermal liquefaction or anaerobic digestion, which can accommodate contaminated feedstocks more safely than direct valorization for food or feed uses. These operational constraints further highlight the importance of selecting downstream valorization routes that are compatible with variable biomass quality and potential contaminant loads [39].

### Atmospheric mitigation and the “energy-carbon” paradox

**Fig. 2:**
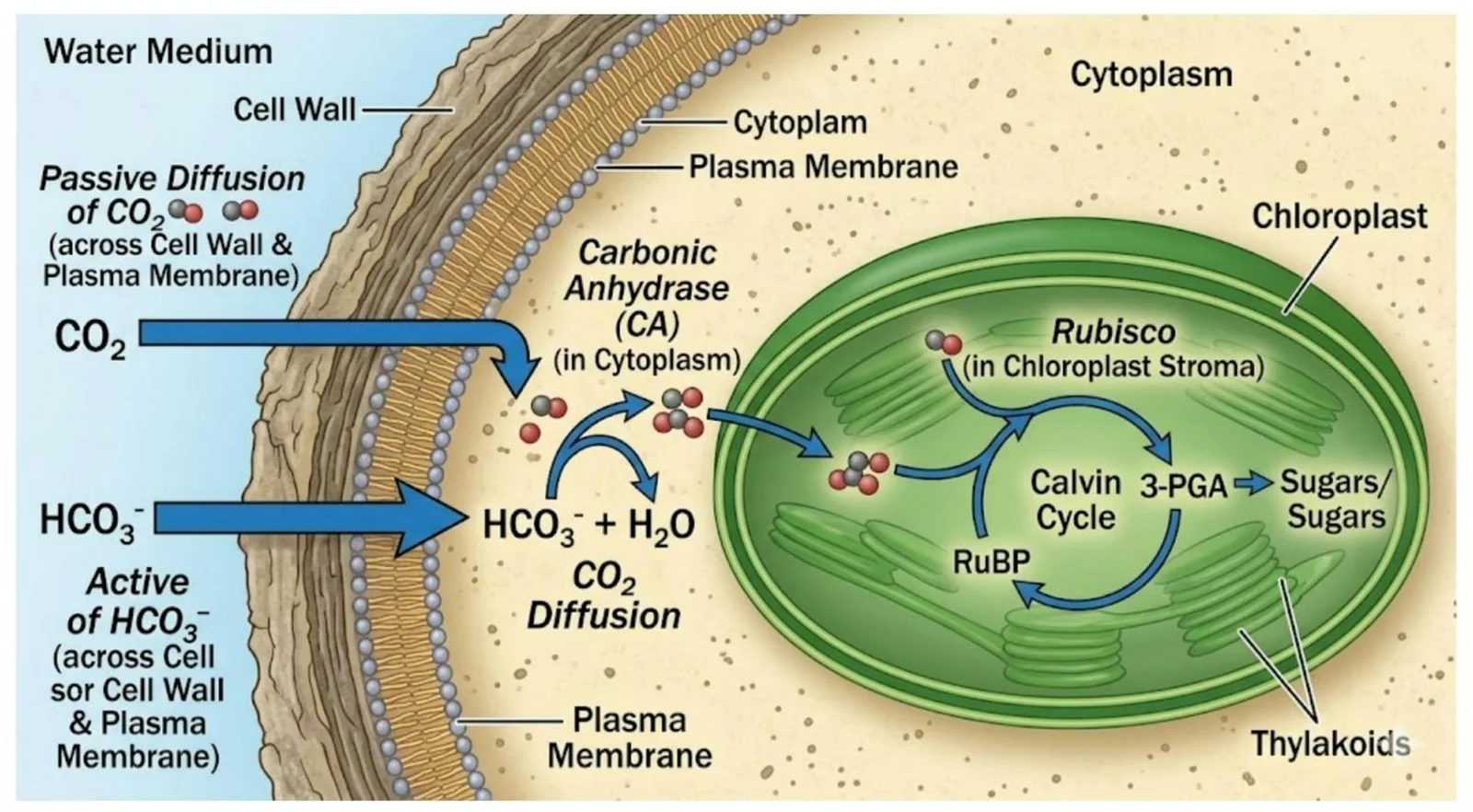
Mass transfer dynamics and enzymatic pathways of the CO₂ Concentrating Mechanism (CCM) for intracellular Carbon dioxide sequestration in microalgae cell factories

A pivotal function of these engineered systems is their capacity for carbon capture, a process fundamentally driven by photosynthesis and CO_2_ sequestration as illustrated in Fig. 2. Microalgae utilize specialized CO_2_-concentrating mechanisms (CCMs) to actively pump inorganic carbon into the carboxysome, allowing them to fix 5.0 × 10⁴ – 1.5 × 10⁵ kg CO₂ ha⁻¹ year⁻¹, a range reported across open-pond and photobioreactor systems for fast-growing species such as *Chlorella* and *Spirulina*, and depends strongly on irradiance, CO₂ delivery efficiency, and areal versus volumetric basis [76]. This represents a 10- to 50-fold efficiency advantage over terrestrial forests, positioning microalgae as an ideal biological “scrubber” for industrial point-source emissions [41-43].

Critical Sustainability Metrics: Despite this potential, the “Net-Zero” claim of algal systems is frequently challenged by the parasitic energy load of upstream operations. The thermodynamic benefit of carbon fixation can be negated by:

- Gas Delivery Dynamics: The power required for micro-bubble sparging to ensure adequate CO_2_ mass transfer and prevent carbon limitation.
- Harvesting Barriers: The “energy trap” of recovery, where the low density of algal cells (typically < 1 g/L in ponds) requires massive energy inputs for centrifugal dewatering and thermal drying.

If these processes are supported by carbon-intensive energy grids, the net environmental footprint may become negligible. Consequently, advancing microalgae as a cornerstone for climate action requires process intensification, specifically the engineering of strains with truncated light-harvesting antennas to reduce shading, and the adoption of circular bioeconomy models that utilize municipal wastewater as a low-cost nutrient source. This holistic integration is necessary to establish microalgae as a technically viable feedstock capable of supporting large-scale industrial waste-to-energy systems.

### Gas-liquid mass transfer limitations

In large-scale microalgae cultivation, the efficiency of carbon delivery is often constrained by gas–liquid mass transfer, particularly the volumetric mass transfer coefficient (*kLa*), which integrates bubble interfacial area and liquid-side resistance [44]. The *kLa* value strongly depends on reactor geometry, sparger design, and gas flow rate; for example, in bubble columns, *kLa* increases linearly with the superficial gas velocity [44], while in flat-panel photobioreactors, micro-bubble spargers can significantly enhance *kLa* and CO₂ capture rates. CO₂ utilization efficiency (the fraction of injected CO₂ actually fixed by microalgae) is directly linked to *kLa* and bubble dynamics. Conventional open raceway ponds typically exhibit low CO₂ utilization efficiencies of only 13–20% owing to rapid outgassing. In contrast, closed photobioreactors can achieve efficiencies exceeding 50% when optimized sparging and pH control are applied. Contemporary strategies, including membrane-based CO₂ delivery and CO₂-absorption enhancers, have further improved mass transfer and productivity. Bubble dynamics plays a pivotal role in these processes: smaller bubbles increase the gas–liquid interfacial area and residence time, thereby improving CO₂ dissolution, but they also require higher energy input for generation. Sparger design therefore involves a trade-off between mass transfer efficiency and operational energy demand. Understanding and optimizing these mass-transfer parameters is crucial for bypassing the scalability trilemma and achieving net-positive CO₂ mitigation in microalgae systems [44].

### Mechanistic insights into carbon partitioning under stress conditions

Beyond bulk CO₂ sequestration, the bioenergy potential of microalgae is fundamentally determined by how photosynthetically fixed carbon is distributed among carbohydrates, lipids, and proteins, a process tightly regulated by nutrient availability and stress signaling. Under nitrogen or phosphorus limitation, microalgae activate a conserved regulatory network involving target of rapamycin (TOR) and sucrose non-fermenting-related kinase (SnRK1) homologs, which repress protein and starch synthesis while derepressing key transcription factors (e.g., bZIP, MYB, and ZnCys families) that drive triacylglyceride (TAG) assembly [45, 46]. Quantitative insights into this redistribution have been obtained through steady-state and dynamic ¹³C-based metabolic flux analysis (MFA). For instance, MFA of *Chlamydomonas reinhardtii* under nitrogen starvation revealed that carbon flux is redirected from the TCA cycle and starch biosynthesis toward de novo fatty acid synthesis, with pyruvate and acetyl-CoA acting as critical branch points [47]. Likewise, flux analysis in *Nannochloropsis oceanica* showed that up to 60% of fixed carbon can be channeled into TAGs under combined phosphorus and nitrogen depletion, primarily through upregulation of diacylglycerol acyltransferase (DGAT) and acetyl-CoA carboxylase (ACCase) [48]. These flux changes are accompanied by post-translational redox regulation of Calvin-cycle enzymes and rerouting of carbon via the oxidative pentose phosphate pathway to supply NADPH for lipid biosynthesis [49]. Integrating transcriptomic and proteomic data with MFA enables the construction of predictive genome-scale metabolic models that guide strain engineering, for instance, by knocking out competing pathways (e.g., starch synthases) or introducing heterologous lipases to boost TAG yields [50]. Understanding these mechanistic underpinnings directly supports the two-stage cultivation strategy widely used in biofuel production: a nutrient-replete growth phase for biomass accumulation, followed by stress-induced metabolic reconfiguration for maximal carbohydrate or lipid yields [51].

### Microalgae biorefineries: processing routes and bioenergy spectrumFF

**Fig. 3:**
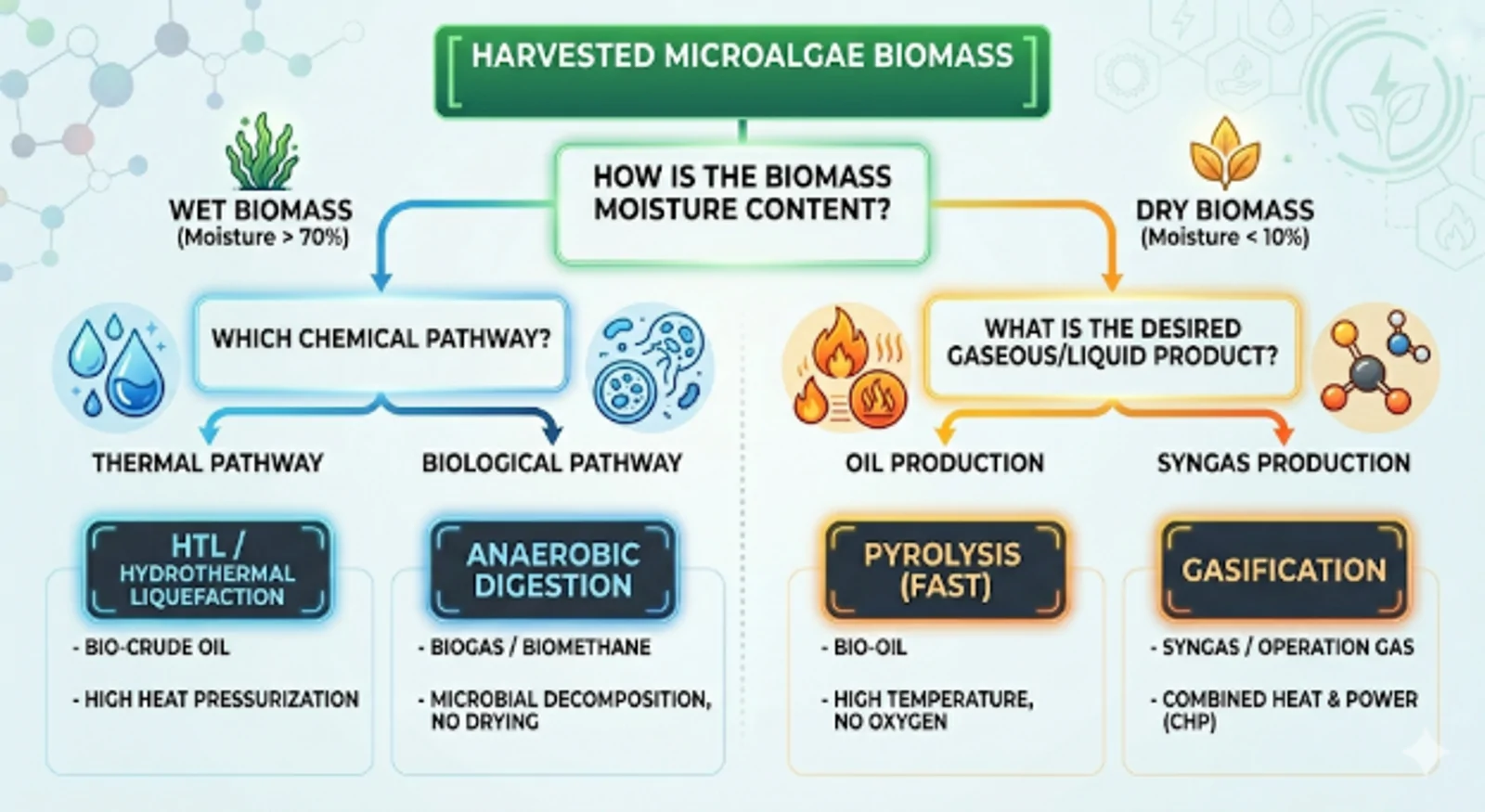
Strategic process-selection matrix for downstream microalgae biomass refining, establishing operational decision pathways based on feedstock moisture content (wt %) and targeted energetic outputs

The conversion of microalgae biomass into bioenergy requires coordinated multi-step processing pathways. Unlike highly structured lignocellulosic feedstocks, microalgae biomass exhibits a highly variable biochemical composition, where lipids, carbohydrates, and proteins coexist within a complex intracellular matrix [51]. Maximizing energy recovery necessitates a transition from standalone technologies to integrated cascade biorefineries, where the thermodynamics of conversion are matched to the specific biochemical signature of the strain (Fig. 3).

#### The biochemical route: beyond simple fermentation

Biochemical pathways represent the “mildest” route of energy extraction, operating under low-temperature regimes that preserve the structural integrity of valuable co-products.

**Fig. 4:**
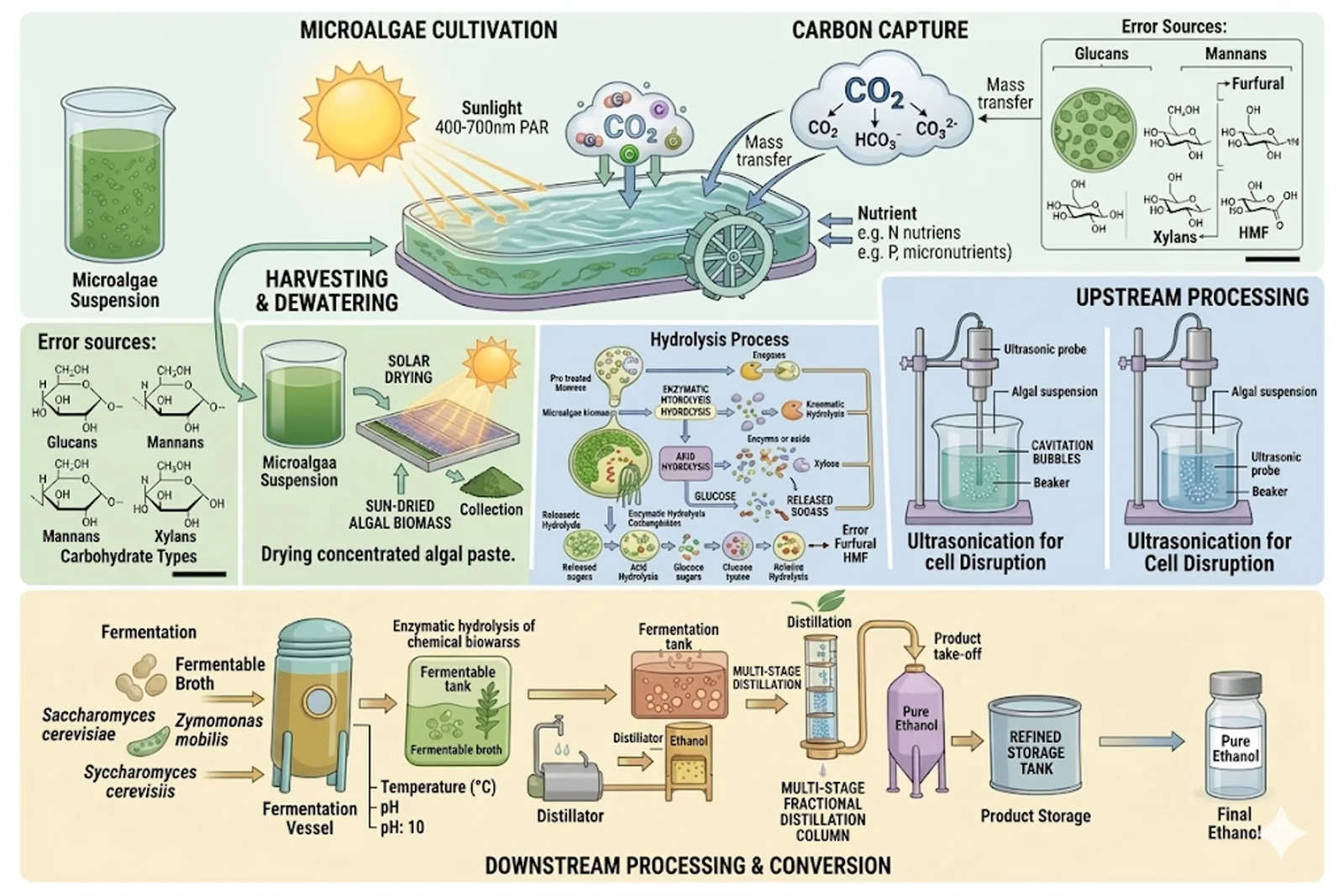
Bioethanol production from microalgae

- Engineered Bioethanol Production: While traditional fermentation targets starch-rich species (e.g., *Chlorococcum humicola*), achieving yields > 500 g/kg (Table 5), the novelty lies in Consolidated Bioprocessing (CBP). By employing engineered microbial consortia, we can synchronize enzyme secretion, saccharification, and fermentation into a single-pot reaction [52]. This integrated approach, as schematically illustrated in Fig. 4, bypasses the “Pretreatment Bottleneck” caused by robust cell walls, minimizing the formation of furan-based inhibitors that typically plague algal ethanol commercialization.

- The Methane-Hydrogen Synergism: Anaerobic Digestion (AD) is evolving from a bulk biogas process into a Two-Stage Bio-Hythane platform. By decoupling the acidogenic phase (targeting H_2_ production) from the methanogenic phase (targeting CH_4_), we can enhance the total energy return (EROI) [53]. The challenge remains the high nitrogen-to-carbon (N/C) ratio of microalgae, which can lead to ammonia toxicity; future systems must utilize Co-digestion with carbon-rich organic wastes to balance the metabolic flux.

#### Thermochemical intensification: high-density energy harvesting

Thermochemical routes (300–1000 °C) provide a “brute force” solution for heterogeneous feedstocks, but the novelty now lies in Hydrothermal Processing [54, 55]. The inherent trade-offs between temperature, pressure, and product yield for each thermochemical route are systematically compared in Table 2, highlighting the shift toward moisture-compatible technologies.

**Table 2:** Comparative analysis of thermochemical conversion processes for algae biomass

Process	Typical Yield	Energy Efficiency	Cost & Operational Factors	Ref.
Pyrolysis	Bio-oil: Up to ~ 47%. Char and gas as co-products.	60–75% (energy recovery as bio-oil + char); endothermic process requiring 2–3 MJ/kg biomass.	**Drying Cost**: 46–46–110/ton product (to < 10% moisture) + **Reactor**: 400–600 °C.	[78]
Liquefaction	Bio-oil: 30–60% wt. (depending on feedstock and conditions).	70–85% (higher heating value basis); advantage: no drying needed for wet feedstocks (80–90% moisture).	**CAPEX**: High-pressure reactor (150–300 bar, 280–370 °C) costs 20–40% more than pyrolysis; **OPEX**: Lower drying cost.	[79]
Gasification	Syngas (CO + H₂ + CH₄): typically 60–75% vol. (H₂ + CO); tar 5–15%.	50–70% (cold gas efficiency); parasitic load for O₂ production if using air separation.	**Drying Cost**: Similar to pyrolysis (46–46–110/ton) + **Cleaning**: Gas cleaning (tar removal) adds 15–30% to plant cost.	[80]

- Hydrothermal Liquefaction (HTL) – The “Wet” Paradigm: HTL is the most promising emerging technology as it utilizes water as both a solvent and a reactant in its sub-critical state. This eliminates the “Energy Trap” of dewatering, converting whole biomass into a premium bio-oil. The research frontier is now focused on In-situ Catalytic Upgrading, where zeolites or noble metal catalysts are integrated into the HTL reactor to reduce the nitrogen and oxygen content of the oil, producing a “drop-in” fuel compatible with existing refineries.
- Advanced Pyrolysis and Gasification: While fast pyrolysis achieves bio-oil yields of ~ 47%, the integration of Slow Torrefaction as a pretreatment step is gaining traction to improve bio-char stability. Gasification, meanwhile, is being reimagined as a source for Bio-Synthetic Natural Gas (Bio-SNG), focusing on plasma-assisted tar remediation to ensure high-purity syngas (CO + H_2_) [56].

#### The chemical and bio-electrochemical vanguard

**Fig. 5:**
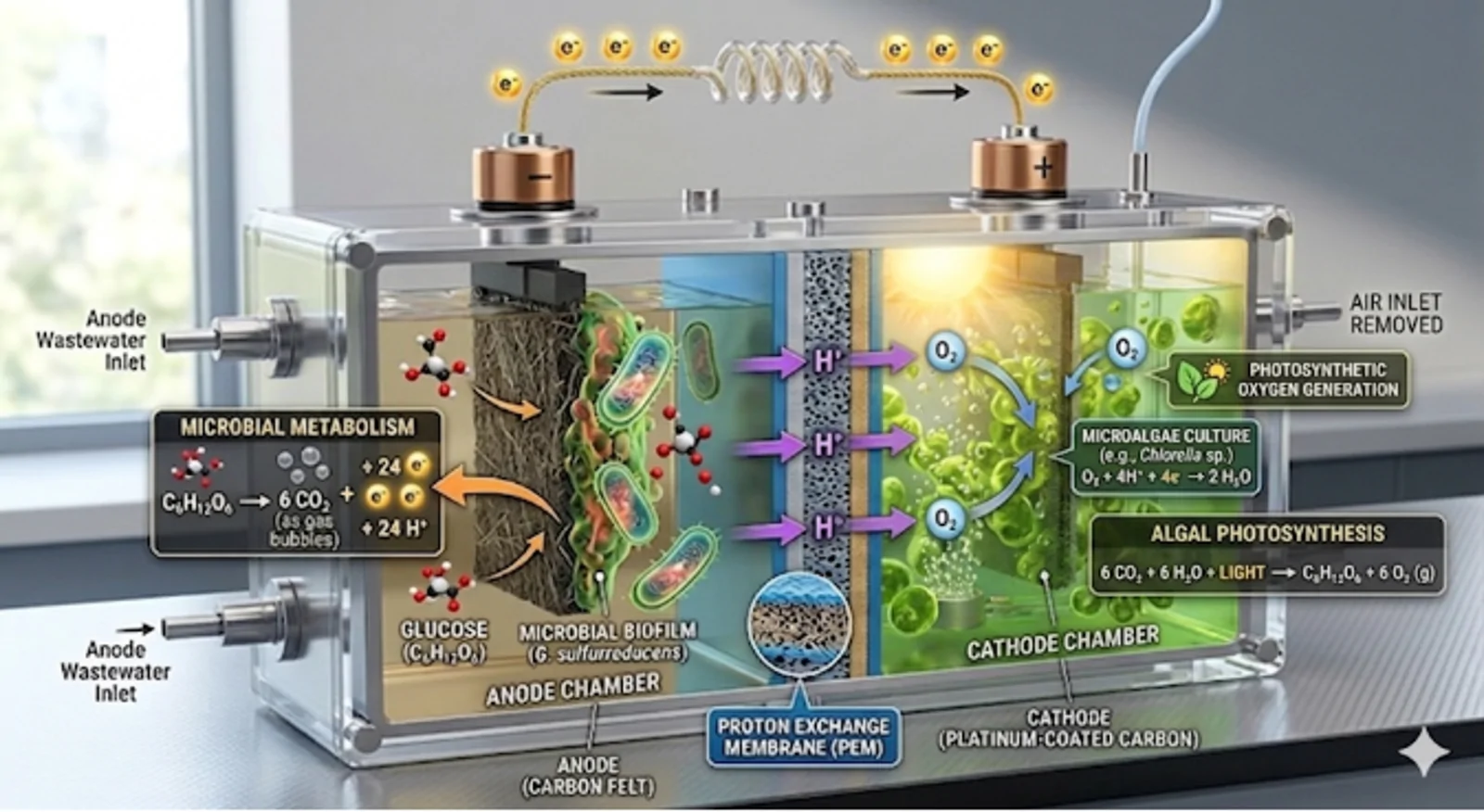
A schematic illustration of a photosynthensytic algae microbial fuel cell

- Transcending Transesterification: Biodiesel production from microalgae represents a strategic alternative to traditional oleaginous crops, offering superior land-use efficiency and lipid yields, as detailed in the comparative analysis in Table 3. While transesterification is a mature field, its novelty is being revived through In-situ Transesterification (Direct Esterification). This method merges lipid extraction and conversion into a single step, drastically reducing solvent recovery costs. Furthermore, the shift toward Enzymatic Lipases, specifically those immobilized on magnetic nanoparticles, allows for catalyst recycling and high-purity glycerol production, turning a waste byproduct into a revenue stream [57].
- Photosynthetic Microbial Fuel Cells (PMFCs): Photosynthetic Microbial Fuel Cells (PMFCs) represent an integrated bio-electrochemical system capable of generating electricity directly from the metabolic pathways of viable microalgae cultures (Fig. 5) [58]. The performance of these systems is highly speffcies-dependent, with various microalgae strains exhibiting distinct electrogenic capabilities, as systematically cataloged in Table 4. The current novelty lies in Bio-photoelectrochemical Cells (BPECs), which utilize the high-energy electrons generated during the early stages of photosynthesis. While power densities remain in the milliwatt range, their role as self-powered “Real-time Biosensors” for monitoring environmental toxicants is a burgeoning niche [34, 59, 65].

**Table 3:** A comparative analysis of microalgae against conventional biodiesel feedstocks

Source	Oil content (% oil by wt. in biomass)	Oil yield (L ha^− 1^ year^− 1^)	Land use (m²·year kg^− 1^ biodiesel)	Biodiesel productivity (kg biodiesel ha^− 1^ year^− 1^)	Ref.
Corn/Maize (*Zea mays L.*)	3–4	150–200	60–70	150–200	[66]
Hemp (*Cannabis sativa L.*)	32–35	350–370	30–32	300–350	[66]
Soybean (*Glycine max L.*)	18–20	400–450	15–20	500–600	[66]
Jatropha (*Jatropha curcas L.*)	30–35	700–800	15–20	650–700	[67]
Camelina (*Camelina sativa L.*)	30–40	500–600	10–15	800–900	[68]
Sunflower (*Helianthus annuus L.*)	35–40	900–1000	10–12	900–1000	[66]
Castor (*Ricinus communis*)	45–50	1300–1500	5–10	1100–1200	[69]
Microalgae (low oil content)	30–40	~ 12,000	0.9–1.1	~ 10,500	[70]
Microalgae (medium oil content)	40–50	~ 20,000	0.6–0.7	~ 17,300	[70]
Microalgae (high oil content)	50–60	~ 25,000	0.5–0.6	~ 22,100	[70]

**Table 4:** Different Microalgae Strains Utilized in Photosynthetic Microbial Fuel Cells (PMFCs)

Microalgae strain	Role	MFC configuration	Power output	Ref.
*Chlamydomonas reinhardtii*	Biocathode	Dual Chamber	15.21 W m^− 2^	[71]
*Synechococcus* sp	Biocathode	Dual Chamber	41.5 m W m^− 2^	[72]
*Chlorella vulgaris*	Biocathode	Dual chamber	126 m W m^− 2^	[73]
*Nitzschia palea* (Diatom)	Bioanode	Dual chamber	12.62 m W m^− 2^	[74]
*Oscillatoria agardhii*	Bioanode (under dark condition)	Dual chamber	26.8 m W m^− 2^	[90]
*Chlorella pyrenoidosa*	Bioanode	Dual Chamber	30.15 m W m^− 2^	[75]

#### Strategic synthesis: the “process lock-in” challenge

The primary bottleneck in energy conversion is no longer the efficiency of a single route, but the “Process Lock-in.” Once a specific pathway is chosen (e.g., dry pyrolysis), it dictates the entire upstream energy expenditure. The future of the field belongs to Dynamic Biorefineries capable of switching outputs based on biomass quality. By routing lipid-extracted residues to AD or gasification, and carbohydrate-rich streams to ethanol or hydrogen, we can achieve near-zero waste metabolism. This holistic integration is the a promising approach to overcome the EROI constraints and position microalgae as a potentially significant contributor. To achieve this, the proposed Circular Bioeconomy Roadmap transcends the ‘Process Lock-in’ challenge by advocating for Dynamic Biorefineries. Unlike traditional linear models, this roadmap integrates upstream engineering with downstream conversion to avoid the ‘Energy Trap’, the massive energy penalty of dewatering and drying. Finally, we clarify how our proposed Circular Bioeconomy Roadmap bypasses these scale-up limitations. By shifting away from standalone systems and instead coupling large-scale cultivation with industrial infrastructures (e.g., utilizing municipal wastewater streams and industrial flue gas), the costs and resources needed for contamination management and mixing energy are offset by “feedstock credits,” turning waste streams into economic drivers for scalable operations. By linking microalgae cell factories directly with industrial infrastructures (flue gas and wastewater), the roadmap transforms waste disposal costs into ‘feedstock credits’, creating a closed-loop system that can improve the prospects for a positive EROI and for commercial scalability under favorable conditions. Realizing these outcomes in practice, however, remains contingent on site-specific factors, including cultivation conditions and geographic location (irradiance, temperature, water availability), the carbon intensity of energy inputs used for mixing, pumping, and gas delivery, the energy demand of harvesting and drying, the maturity and efficiency of the chosen conversion technology, and the market value achievable for co-products. The roadmap should therefore be understood as identifying the structural conditions under which positive EROI and commercial scalability become achievable, rather than as a guarantee that they will be achieved in any given deployment.

### Technology readiness level (TRL) mapping of advanced bioenergy pathways

The previous sections have described several advanced conversion concepts that range from well-established unit operations to highly innovative, emerging strategies. To avoid conflating speculative research directions with near-term deployable technologies, this subsection explicitly classifies each major concept according to standard Technology Readiness Level (TRL) definitions adapted from Horizon Europe.

TRL definitions used here:

- TRL 2–3: Technology concept formulated / experimental proof of concept demonstrated at laboratory scale.
- TRL 4–5: Technology validated in laboratory environment or relevant environment (small pilot).
- TRL 6–7: Technology demonstrated in relevant environment (large pilot) or system prototype qualified in operational environment.
- TRL 8–9: System complete and qualified through test and demonstration / actual system proven in operational environment.

Table 5 summarizes the TRL assessment, key literature evidence, and principal gaps for each advanced technology discussed in this review.

**Table 5:** TRL Mapping of Advanced Microalgae Bioenergy Concepts

Technology / Concept	Technology Readiness Level (TRL)	Current Status & Evidence	Key Gap
Designer strains (e.g., high-lipid *Chlorella*, Clustered Regularly Interspaced Short Palindromic Repeats (CRISPR)-engineered)	4–6	Lab and pilot-scale demonstrations exist; some strains tested outdoors.	Regulatory and containment challenges for open systems.
Consolidated bioprocessing (CBP) for bioethanol	2–3	Proven in vitro with engineered consortia; no scaled microalgal system.	Enzyme stability and inhibitor formation remain unsolved.
Two-stage bio-Hythane (H₂ + CH₄)	5–6	Pilot‑scale two‑stage anaerobic digestion validated (e.g., mixed algae + waste).	Needs long‑term stability and gas composition control.
In-situ catalytic HTL upgrading	3–4	Bench-scale with zeolites/noble metals; improves bio-oil quality.	Catalyst deactivation, cost, and scale-up not yet shown.
Dynamic biorefinery (switchable output)	2–3	Conceptual and optimization models exist; no physical demonstration.	Requires real-time biomass monitoring and flexible unit ops.
Photosynthetic microbial fuel cells (PMFCs)	3–4	Power densities in mW range; 120 L pilot reported; niche use as biosensors.	Power output too low for grid; biosensing more plausible.

Technology Readiness Levels (TRLs) were classified according to the European Commission’s Horizon Europe definition, where TRL 1–3 correspond to basic principles observed, technology concept formulated, and experimental proof of concept; TRL 4–5 to validation in laboratory and relevant environments; TRL 6–7 to demonstration and prototype testing in relevant/operational environments; and TRL 8–9 to system completion and proven operational or commercial maturity [92, 94]

## The techno-economic and environmental (TEE) nexus: beyond isolated metrics

The transition of microalgae from a laboratory concept to an industrial reality is governed by a complex “TEE Nexus,” where financial viability and ecological integrity are inextricably linked. Commercial success depends not only on high lipid productivity (in select oleaginous species such as *Botryococcus braunii* and *Nannochloropsis* sp.) under nutrient-stressed laboratory or pilot-scale photobioreactor conditions, which can exceed 70% DCW when subjected to specific cultivation stresses tailored for biodiesel optimization, but also on a multidisciplinary strategy that dissolves existing bottlenecks across the value chain [60, 62].

### The economic-environmental dualism

Current Life Cycle Assessments (LCA) and Techno-Economic Analyses (TEA) reveal a dual narrative. On one hand, microalgae offer significant environmental benefits: fixing 5.0 × 10⁴ – 1.5 × 10⁵ kg CO₂ ha⁻¹ year⁻¹ and providing tertiary wastewater treatment by stripping nitrogen and phosphorus . However, these benefits are often offset by the “Energy Trap”, the high fossil fuel inputs required for hydraulic pumping, thermal drying, and centrifugal harvesting [64].

To achieve a positive Energy Return on Investment (EROI), the industry must shift toward “low-energy pathways,” such as heterotrophic cultivation in conventional bioreactors [15]. This approach, while sacrificing photosynthetic carbon capture, drastically simplifies reactor design and stabilizes production costs, often resulting in a carbon footprint that is competitive with petroleum-based standards [65].

To make these trade-offs directly comparable across major cultivation and conversion approaches, Table 6 summarizes typical reported ranges for energy consumption, biomass or product production cost, greenhouse gas (GHG) emissions, water use, productivity, and the system boundary assumed in each case. The wide ranges reflect genuine variability in the underlying literature (geography, scale, and system design) rather than measurement imprecision, and system boundaries are stated explicitly because failing to align them is a common source of apparently contradictory results in the microalgae LCA/TEA literature.

**Table 6:** Comparative summary of typical reported energy consumption, production cost, GHG emissions, water use, productivity, and system boundary for major microalgae cultivation and conversion approaches. Ranges are illustrative of literature-reported variability rather than a single deterministic estimate

Approach	System boundary	Energy consumption	Production cost US$	GHG emissions	Water use	Productivity	Ref.
Open raceway pond cultivation	Cradle-to-pond-gate (cultivation + harvesting)	~ 5–15 MJ kg⁻¹ biomass	~ 3.7–6.3 kg⁻¹ biomass	~ 2–4 kg CO₂-eq kg⁻¹ biomass (before any credits)	~ 500–3000 L kg⁻¹ biomass (net, evaporative losses)	10–25 g m⁻² d⁻¹ (areal)	[85, 86]
Closed photobioreactor cultivation	Cradle-to-reactor-gate (cultivation + harvesting)	~ 15–40 MJ kg⁻¹ biomass (mixing, cooling, artificial light where used)	~ 8–20 kg⁻¹ biomass	~ 4–8 kg CO₂-eq kg⁻¹ biomass	~ 200–500 L kg⁻¹ biomass (largely recirculated)	1–4 g L⁻¹ d⁻¹ (volumetric)	[85, 87]
Wastewater-fed cultivation	Cradle-to-pond/reactor-gate, wastewater-treatment credit included	~ 2–8 MJ kg⁻¹ biomass (nutrients supplied by wastewater)	~ 2–5 kg⁻¹ biomass	Net-negative to + 1 kg CO₂-eq kg⁻¹ biomass (treatment credit dependent)	~ 0 net freshwater (uses wastewater as primary water source)	10–20 g m⁻² d⁻¹ (areal)	[88, 89]
Transesterification (biodiesel conversion)	Gate-to-gate (extraction + conversion only)	~ 5–10 MJ kg⁻¹ oil processed	Add ~ 1–3 kg⁻¹ to feedstock cost	~ 1–3 kg CO₂-eq kg⁻¹ biodiesel	Low (largely process water, solvent-dependent)	Not applicable (conversion step)	[90, 91]
Anaerobic digestion (biogas/bio-hythane)	Gate-to-gate (digestion of residual/whole biomass)	~ 1–3 MJ kg⁻¹ biomass processed	Low relative capital cost per unit throughput	Net-negative to low-positive (avoided waste-treatment emissions, methane credit)	Largely recycled as digestate/process water	Not applicable (conversion step)	[90]
Hydrothermal liquefaction (HTL)	Gate-to-gate (wet-biomass conversion, no drying required)	~ 10–20 MJ kg⁻¹ biomass (lower than routes requiring drying)	Pilot-stage; not yet consistently reported at commercial scale	~ 2–5 kg CO₂-eq kg⁻¹ bio-crude	Low incremental (uses biomass slurry directly)	Not applicable (conversion step)	[92,93]

GHG, greenhouse gas; MJ, megajoule; kg CO₂-eq, kilograms of carbon-dioxide equivalent; L, litre; g m⁻² d⁻¹, grams per square metre per day (areal productivity, cultivation rows only); g L⁻¹ d⁻¹, grams per litre per day (volumetric productivity, closed-photobioreactor row only); US$ and €, United States dollars and euros respectively, reported in the currency and publication year of the source. “Not applicable” in the productivity column indicates a conversion-stage process for which an areal or volumetric cultivation productivity is not a meaningful metric

### Resolving the “scalability trilemma*”*

The primary hurdle to large-scale deployment is a fundamental trilemma: the conflict between low CAPEX (favoring open ponds), high process control (favoring photobioreactors - PBRs), and minimal environmental footprint. To weave these operational challenges together cohesively, we have integrated these dynamics into our revised ‘Scalability Trilemma’ framework. This structural framework systematically weighs the complex trade-offs between the low CAPEX of open ponds, the precise process control and strict biosecurity of closed PBRs, and critical environmental sustainability metrics like water footprints and net carbon capture efficiency.

- The Resource Constraint: While open ponds are economically attractive, their high evaporative water footprint and vulnerability to contamination limit their reliability.
- The Policy Gap: In the absence of robust carbon pricing, algal biofuels struggle to compete with subsidized fossil incumbents. Commercial viability, therefore, is not a standalone goal but a factor of “Symbiotic Industrial Ecosystems”, linking algae production directly with industrial flue gas and municipal waste streams to turn “disposal costs” into “feedstock credits.”

### Strategic roadmap: toward a circular biofactory

To systematically assess technological maturity across the microalgal valorization pathway, Technology Readiness Levels (TRLs) were evaluated on a 1–9 scale based on the European Commission (Horizon Europe) framework. The TRL classifications reported herein are synthesized from published techno-economic analyses, pilot-plant reports, and commercial deployment benchmarks [66, 67] Technologies at TRL 1–3 represent basic conceptual or laboratory validation; TRL 4–6 indicate pilot-scale or simulated environment demonstration; and TRL 7–9 denote industrial-scale operation or full commercial deployment (Table 5). To transcend incremental improvements, a transformative roadmap focusing on “Circular Bioeconomy Platforms” is essential. Consistent with the TRL classifications in Table 5, the three elements of this roadmap differ substantially in maturity: some components are already demonstrated at pilot or commercial scale and represent near-term deployment opportunities, while others remain at the laboratory or conceptual stage and constitute medium- to long-term research priorities. Each element below is labeled accordingly.

A. Eco-Physiological Engineering: Future strain development must move beyond single-trait optimization (e.g., just lipids) toward “Designer Strains.” These should feature inducible cell lysis for energy-efficient harvesting and the ability to thrive in untreated, fluctuating waste streams. Maturity: laboratory/conceptual stage (TRL 3–4, consistent with CRISPR/Cas-based strain engineering in Table 5); this is a medium- to long-term research priority rather than a near-term deployment option.
B. Modular Biorefining: Research must pivot toward low-temperature catalytic refining and electrochemical upgrading. By adopting a “cascade” approach, where high-value proteins and pigments are extracted before fuel conversion, the economic burden is distributed across multiple revenue streams. Maturity mixed): The cascade extraction of proteins and pigments ahead of fuel conversion is already practiced commercially for high-value products (TRL 7–9) and is near-term deployable; the low-temperature catalytic refining and electrochemical upgrading of the residual fraction remain at laboratory to small-pilot scale (TRL 3–5, consistent with the HTL and enzymatic-route entries in Table 5) and represent a medium-term research priority.
C. Regional Hubs: Future deployment should be guided by “Dynamic Eco-Economic Models” that identify optimal geographic hubs. These hubs would integrate algae cultivation with existing power plants and wastewater utilities, creating a self-sustaining industrial metabolism. Maturity (mixed): Co-locating cultivation with flue-gas and wastewater sources has been demonstrated at pilot and demonstration scale (TRL 5–6, consistent with the wastewater-fed cultivation entry in Table 5) and is realistic for near- to medium-term deployment; the formal “Dynamic Eco-Economic Model” for systematically siting such hubs is, to our knowledge, a conceptual planning framework that has not yet been operationalized or validated against real deployment data, and should be regarded as a research and decision-support priority rather than an established siting tool.

## Conclusion

This review frames the microalgae-based bioenergy value chain as an integrated circular-bioeconomy system, not a set of isolated technologies. Technologically, it spans a wide maturity spectrum: open-pond cultivation (TRL 8–9), closed photobioreactors (TRL 6–7), and commercial-scale cascade extraction of high-value co-products are well-established, whereas CRISPR/Cas engineering, defined consortia, advanced conversion routes (e.g., consolidated bioprocessing, two-stage anaerobic digestion), and photosynthetic microbial fuel cells remain at early pilot stages (TRL 2–5). Crucially, techno-economic and environmental performance is highly context-dependent; biomass costs, energy use, emissions, and water footprint vary by an order of magnitude based on system design, location, energy sources, and co-product markets. The so-called “Energy Trap”, stemming from pumping, drying, and harvesting, means that a net-positive energy return is conditional, not intrinsic, and commercial scalability hinges on a confluence of site-specific factors rather than any single breakthrough. Consequently, realizing microalgae’s circular-bioeconomy potential demands coordinated, incremental, and location-tailored progress. The review cautions against extrapolating laboratory or pilot successes to universal commercial viability. It underscores that transparent reporting of system boundaries, cultivation variables, and co-product valorization, alongside targeted Research and development (R&D), remains essential to bridge the gap between theoretical potential and deployed reality.

## Data Availability

No datasets were generated or analysed during the current study.

## Abbreviations

AD: Anaerobic digestion
CCM: CO₂ concentrating mechanism
CRISPR: Clustered regularly interspaced short palindromic repeats
DCW: Dry cell weight
EPA: Eicosapentaenoic acid
EROI: Energy return on investment
GHG: Greenhouse gas
HTL: Hydrothermal liquefaction
LCA: Life cycle assessment
MFC: Microbial fuel cell
PBR: Photobioreactor
PMFC: Photosynthetic microbial fuel cell
TAG: Triacylglycerides
TEA: Techno-economic analysis
TEE: Techno-economic-environmental
TRL: Technology readiness level
TOR: Target of rapamycin
SnRK1: Sucrose non fermenting related kinase
